# Evaluating Tibial Tunnel Landmarks in Anterior Cruciate Ligament Reconstruction: Remnant Versus Lateral Meniscus Anterior Horn

**DOI:** 10.3390/jcm14228096

**Published:** 2025-11-15

**Authors:** Gokhan Pehlivanoglu, Kadir Ilker Yildiz, Kutalmis Albayrak, Tolga Cakir, Umit Selcuk Aykut, Baris Ozkul

**Affiliations:** 1 Department of Orthopaedics and Traumatology, University of Health Sciences Baltalimani Bone Diseases Training and Research Hospital, 34470 Istanbul, Turkey; 2 Department of Orthopaedics and Traumatology, Amasya Suluova State Hospital, 05500 Amasya, Turkey

**Keywords:** ACL reconstruction, ACL remnant, intraoperative landmarks, lateral meniscus anterior horn, tibial tunnel placement

## Abstract

**Background:** We sought to compare the anatomical accuracy and clinical outcomes of two intraoperative landmarks, the anterior cruciate ligament (ACL) tibial remnant and the anterior horn of the lateral meniscus (LMAH), for tibial tunnel placement in single-bundle ACL reconstruction. **Methods:** This retrospective comparative study included 74 patients who underwent single-bundle ACL reconstruction using either the ACL tibial remnant (Group 1, *n* = 35) or the LMAH (Group 2, *n* = 39) as the primary intraoperative landmark. Tunnel positioning was evaluated using post-operative MRI. Clinical outcomes included the Lysholm score, subjective IKDC, Tegner activity scale, Lachman and pivot-shift tests, KT-2000 arthrometer measurements, and return-to-sports status. **Results:** Group 1 demonstrated slightly more anterior sagittal tunnel placement (44.57%) compared to Group 2 (46.87%) (*p* = 0.036). Coronal tunnel positioning did not differ significantly between the groups (*p* = 0.215). Functional scores, stability tests, and return-to-sports rates were similar across groups. MRI-based tunnel measurements in sagittal and coronal planes had excellent inter- and intraobserver reliability. **Conclusions:** Both the ACL tibial remnant and LMAH are reliable landmarks for tibial tunnel placement in ACL reconstruction. Although minor variations in sagittal tunnel positioning were identified, they did not affect functional or stability outcomes.

## 1. Introduction

Anterior cruciate ligament (ACL) reconstruction is a widely performed procedure to restore knee stability after rupture [1,2,3]. Accurate positioning of the tibial tunnel is critical to restore normal knee kinematics and prevent complications such as graft impingement or residual instability [1,4]. Several anatomical landmarks have been proposed to guide tunnel placement, including the posterior cruciate ligament, medial tibial eminence, anterior horn of the lateral meniscus (LMAH), and the ACL tibial remnant [5].

The ACL remnant is often preserved and serves as a practical intraoperative reference for identifying the native insertion site [1]. However, its visibility and morphology can vary, particularly in chronic injuries or after prior debridement [6,7]. Recent histological observations suggest that these differences may be related to variations in the surrounding connective tissue (epiligament), which influence the vascularity and cellularity of the remnant [8,9,10,11]. This biologic variability may help explain the inconsistent structural quality of the ACL remnant encountered during surgery.

The LMAH, on the other hand, is consistently identifiable during arthroscopy, although its spatial relationship with the ACL footprint may vary between individuals [12,13].

Despite the frequent use of both landmarks, evidence remains limited regarding their accuracy in achieving anatomical tunnel placement or their impact on clinical outcomes. To our knowledge, this is the first study to radiologically and clinically compare the ACL tibial remnant and the LMAH as landmarks for tibial tunnel placement in single-bundle ACL reconstruction.

This study aimed to evaluate and compare the anatomical accuracy and functional outcomes of tibial tunnels created using the ACL remnant versus the LMAH. We hypothesized that the ACL remnant might result in slightly more anterior sagittal tunnel placement, but that both landmarks would yield comparable functional results.

## 2. Materials and Methods

### 2.1. Study Design and Participants

This retrospective comparative study was approved by the Institutional Review Board (IRB) of Metin Sabancı Baltalimanı Bone Diseases Training and Research Hospital (Approval No: 36-277/Date: 14 May 2025). All procedures performed in this study involving human participants were conducted in accordance with the ethical standards of the 1964 Helsinki Declaration and its later amendments. Patients who underwent arthroscopic single-bundle ACL reconstruction using hamstring autografts between 2019 and 2023 were retrospectively reviewed. Inclusion criteria were isolated ACL rupture, availability of post-operative magnetic resonance imaging (MRI) scans within the institutional system, and patients with closed physes. Patients with concomitant injuries (e.g., meniscal tears, cartilage lesions, collateral ligament injuries) or a history of previous knee surgery were excluded.

### 2.2. Surgical Groups

All procedures were performed by orthopedic surgeons experienced in ACL reconstruction [14]. Two surgeons (K.I.Y. and K.A.) consistently used the ACL tibial remnant as the primary intraoperative landmark (Group 1, *n* = 35), whereas two others (G.P. and U.S.A.) used the LMAH (Group 2, *n* = 39).

### 2.3. Surgical Technique

All procedures were performed under spinal anesthesia with the patient in the supine position using a standard arthroscopic technique. Diagnostic arthroscopy was first conducted to confirm isolated ACL rupture and rule out associated injuries. A hamstring tendon autograft was harvested via an anteromedial oblique incision and prepared as a quadrupled graft.

The femoral tunnel was created via an anteromedial portal technique, with femoral fixation achieved using a cortical suspensory device (Liftfix button; Tulpar, Ankara, Turkey). Tibial fixation was performed using a bioabsorbable interference screw (Tulpar, Ankara, Turkey) supplemented with a staple for added strength.

Tibial tunnel guidewire placement was based on the surgeon’s preferred intraoperative landmark:

- Group 1 (ACL remnant group): The surgeon used the ACL tibial remnant as the primary reference. To identify the center of the remnant, partial debridement was performed and standardized using a scaled arthroscopic probe with a 4-mm hook length (1-mm markings), leaving ~5 mm of tissue. The guidewire was then inserted through the central portion of the remnant.
- Group 2 (LMAH group): Following complete debridement of the ACL remnant, surgeons used the posterior margin of the LMAH as a reference. The guide wire was aligned mediolaterally with the center of the intercondylar eminence (Note: The same tibial aiming device was used in both groups).

Tibial tunnels were reamed over the guidewire according to the graft diameter. After graft passage and tensioning, fixation was completed as described above.

### 2.4. Post-Operative Rehabilitation

All patients followed a standardized institutional rehabilitation protocol. A hinged knee brace was applied for the first four weeks. Weight-bearing was initiated at four weeks, while knee flexion was restricted to 90° during the first three weeks to protect graft integrity. Return to sports was not permitted until at least six months post-operatively.

### 2.5. Imaging and Measurement

**Figure 1:**
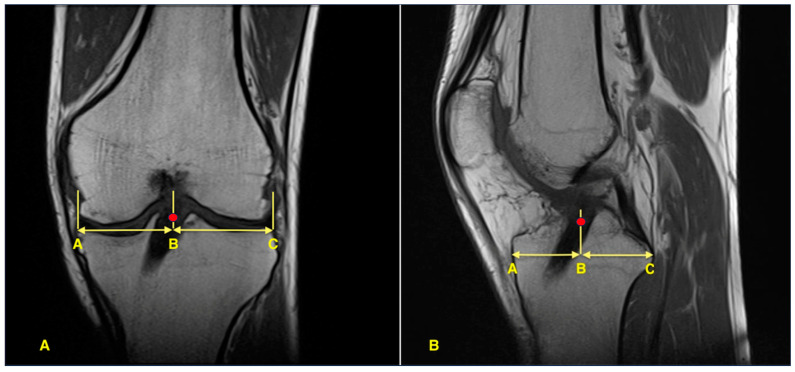
Measurement of tibial tunnel position on post-operative MRI. The red dot indicates the center of the graft. (**A**) Coronal view showing mediolateral tunnel position calculated as the percentage ratio AB/AC. (**B**) Sagittal view showing anteroposterior tunnel location calculated as the percentage ratio AB/AC.

Post-operative MRI scans were obtained at 12 months after surgery. All MRIs were performed using a 1.5-T scanner (MAGNETOM Symphony; Siemens AG, Erlangen, Germany) with 3-mm slice thickness, including standard knee protocols (sagittal and coronal proton density–weighted sequences with fat suppression). Tibial tunnel positions were evaluated on sagittal and coronal images. Sagittal tunnel location was measured as a percentage of the anteroposterior tibial plateau length based on the method described by Stäubli and Rauschning [15], while coronal tunnel position was calculated as a percentage of the mediolateral tibial plateau width as described by Agneskirchner et al. [16] (see Figure 1).

All measurements were independently performed by two orthopedic surgeons who were blinded to the surgical groups. To assess interobserver reliability, both observers measured all cases at baseline. For intraobserver reliability, one observer repeated the measurements after a 45-day interval. Intraclass correlation coefficients (ICCs) were calculated to determine both interobserver and intraobserver agreement. ICC values were interpreted as follows: <0.50 poor reliability, 0.50–0.75 moderate, 0.75–0.90 good, and >0.90 excellent reliability [17].

### 2.6. Data Collection

Baseline demographic data including age, sex, height, weight, and operated side, were recorded. Functional outcomes were assessed using the Lysholm score, Tegner activity scale, and subjective IKDC (International Knee Documentation Committee) score. Knee stability was assessed using the Lachman and pivot-shift tests. Anterior laxity was quantified as the side-to-side difference in displacement using the KT-2000 Arthrometer (Medmetric Corp., San Diego, CA, USA) under manual maximum force at 30° of knee flexion.

Patients were also surveyed regarding returning to sports. They were asked whether they had resumed sporting activities (yes/no), and if so, whether they returned at a lower, the same, or a higher level compared to preinjury status. The type of sport (pivoting or non-pivoting) and the time to return to sports (months) were also recorded.

Graft failure was evaluated based on clinical evidence of recurrent instability and MRI confirmation of graft discontinuity.

### 2.7. Statistical Analysis

The primary outcome variable was the sagittal tibial tunnel position, expressed as the percentage of the anteroposterior (AP) tibial plateau length measured by the Stäubli–Rauschning method.

A priori power analysis (G*Power Version 3.1.9.6; Heinrich Heine University Düsseldorf, Düsseldorf, Germany) based on the effect size reported by Büyükdoğan et al. [18] for tibial tunnel placement ratio (Cohen’s d = 3.05, α = 0.05, power = 0.95) indicated that a minimum of five patients per group would be sufficient to detect a significant difference. The final cohort met this threshold, ensuring sufficient statistical power to support the validity of the study findings.

All analyses were performed using IBM SPSS Statistics Version 27.0.1.0 (IBM Corp., Armonk, NY, USA). Descriptive statistics were presented as mean ± standard deviation (SD) for continuous variables and as frequency (percentage) for categorical variables. The normality of the continuous variables was assessed using the Shapiro–Wilk test.

Between-group comparisons were made using the independent samples t-test for normally distributed variables or the Mann–Whitney U test for non-normal data. Categorical variables were compared using the chi-square test or Fisher’s exact test, as appropriate. Pre- and post-operative changes within groups were assessed using the Wilcoxon signed-rank test for ordinal variables and paired t-test or Wilcoxon test for continuous variables depending on data distribution.

For variables measured at three time points (preinjury, preoperative, and postoperative), the Friedman test was used to compare repeated measurements within each group. When the Friedman test indicated statistical significance, pairwise post hoc analyses were performed using the Wilcoxon signed-rank test with Bonferroni correction to adjust for multiple comparisons (*p* < 0.017 considered significant).

Intraclass correlation coefficients (ICCs) were calculated to evaluate inter- and intraobserver reliability using a two-way random-effects model with absolute agreement for single measures.

For secondary outcomes, multiple comparisons were interpreted descriptively, and statistical significance was set at a two-tailed *p* < 0.05 for all analyses, except for repeated-measures tests (e.g., Tegner activity scale), where an adjusted threshold of *p* < 0.017 was applied.

## 3. Results

Seventy-four patients were included in the study, with 35 patients in Group 1 (ACL remnant) and 39 patients in Group 2 (LMAH). There were no statistically significant differences between the groups regarding demographic characteristics (Table 1).

Functional outcomes improved significantly from pre-operative to post-operative assessments across the groups. For the Tegner activity scale, a significant difference was detected among preinjury, preoperative, and postoperative scores according to the Friedman test (*p* < 0.001). Post hoc pairwise analyses revealed a significant decrease from preinjury to preoperative status (*p* < 0.001) and a subsequent improvement from preoperative to postoperative (*p* < 0.001), while postoperative scores remained lower than preinjury levels (*p* < 0.01). Comparable significant improvements were observed in Lysholm knee and IKDC scores (both *p* < 0.001). Between-group comparisons revealed no statistically significant differences in Tegner scores at any time point (preinjury, preoperative, or postoperative), or in Lysholm and IKDC scores (all *p* > 0.05) (Table 2).

Stability tests also demonstrated significant post-operative improvement. Both instrumented laxity and pivot shift scores improved significantly within each group (*p* < 0.001, Wilcoxon signed-rank test). However, no statistically significant differences were found between Group 1 and Group 2 at either the pre-operative or post-operative time points for Lachman (preop *p* = 0.587; postop *p* = 0.856) or pivot-shift scores (preop *p* = 0.496; postop *p* = 0.799) (Mann–Whitney U test). KT-2000 arthrometer measurements revealed no significant difference between the groups (Group 1: 2.03 ± 1.48 mm, Group 2: 2.18 ± 1.94 mm; *p* = 0.978, Mann–Whitney U test) (Table 3).

Return-to-sports analysis showed that 30 of 35 patients (85.71%) in Group 1 and 32 of 39 patients (82.05%) in Group 2 were able to return to sports post-operatively (*p* = 0.759). Most of these patients participated in pivoting sports (Group 1: 23/30; Group 2: 25/32), while 7 patients in each group performed non-pivoting activities. All patients who were unable to return to sports had previously participated in pivoting sports. Regarding the level of return, four patients in Group 1 and six in Group 2 reported returning at a lower level than preinjury status, while 26 patients in each group returned at the same level. No patients returned at a higher level in either group (*p* = 0.733) The mean time to return to sports was 8.83 ± 2.17 months in Group 1 and 8.88 ± 1.90 months in Group 2 (*p* = 0.791) (Table 4).

Radiographic analysis of the tibial tunnel position using the Stäubli and Rauschning method indicated that the sagittal tunnel position was significantly more posterior in Group 2 compared to Group 1 (*p* = 0.036; Cohen’s d = 0.50, 95% CI 0.03–0.96) [15]. Similarly, the anterior cortex to graft center distance differed significantly between the groups (*p* = 0.038; Cohen’s d = 0.49, 95% CI 0.02–0.96), while anterior cortex to posterior cortex values were comparable (*p* = 0.466). No significant difference was found in any of the coronal plane tunnel positioning parameters between groups (all *p* > 0.05) (Table 5).

A total of five graft failures were observed (Group 1 *n* = 2; Group 2 *n* = 3) whereas no statistical significancy (*p* = 1.000) was seen.

Interobserver reliability for sagittal tunnel position measurements was excellent, with an ICC of 0.970 (95% CI, 0.952–0.981) at baseline. Intraobserver reliability (same observer, baseline vs. day 45) was also excellent, with an ICC of 0.979 (95% CI, 0.967–0.987). For coronal measurements, interobserver and intraobserver ICCs were 0.932 (95% CI, 0.894–0.957) and 0.939 (95% CI, 0.904–0.961), respectively [17].

## 4. Discussion

The primary finding of this study was a statistically significant difference in sagittal tibial tunnel placement between the two groups, as assessed by post-operative MRI. The tibial remnant group exhibited a slightly more anterior tunnel placement, with a mean sagittal placement of 44.57% compared to 46.87% in the LMAH group (*p* = 0.036), corresponding to an approximate shift of 1.5 mm in the sagittal plane (24.69 ± 3.23 mm vs. 26.20 ± 2.93 mm, *p* = 0.038). However, this radiological difference was not associated with any statistically significant differences in clinical outcomes. Additionally, no significant difference was detected between the groups in terms of coronal tunnel placement, with mean values of 47.99% for Group 1 and 48.77% for Group 2 (*p* = 0.215).

Numerous studies have emphasized the critical importance of tunnel placement in ACL reconstruction. Improper tibial tunnel positioning has been associated with specific complications: anterior placement may cause roof impingement, while posterior placement has been linked to increased rotational instability [5,12]. Thus, accurate tunnel positioning requires a precise understanding of the native ACL anatomy, which has been extensively explored through cadaveric and anatomical investigations [15,19].

In a cadaveric study, Stäubli and Rauschning localized the center of the ACL tibial footprint at 43.3% along the sagittal axis of the tibial plateau and recommended targeting the 44% position for optimal tunnel placement [15]. Similarly, Parkar et al. [19], in a systematic review analyzing 300 knees, reported a mean footprint position of 42%, with a 5th to 95th percentile range centered around 44%. These findings have established the 44% anteroposterior reference point as the anatomical benchmark for tibial tunnel placement.

Clinical studies confirm these anatomical benchmarks in vivo. Silva et al. reported tibial tunnel centers at 44.4% in the transportal group and 55.4% in the transtibial group [20]. Yau et al. similarly found centers at 47% and 51%, respectively [21]. Our findings align well with these data: using the transportal technique, mean placement was 44.57% when referenced to the ACL remnant and 46.87% using the LMAH landmark, supporting the clinical utility and anatomical relevance of these intraoperative reference points for accurate tibial tunnel placement.

The posterior border of the LMAH was first described by Jackson and Gasser as a reliable, easily identifiable landmark for tibial tunnel placement [12] and remains one of the most widely utilized reference points in clinical practice. Kassam et al. [22] radiologically validated this landmark, demonstrating its posterior border lies on average only 0.1 mm posterior to the midpoint of the ACL tibial footprint, reinforcing its precision as a sagittal plane reference. Dimitriou et al. further identified the LMAH and medial tibial spine (MTS) as useful alternative landmarks, especially in revision cases without an ACL remnant [23].

However, anatomical investigations such as those by Ferretti et al. have revealed that the LMAH does not maintain a universally constant spatial relationship to the ACL tibial insertion, underlining notable inter-individual variation [13]. Clinical and intraoperative studies, including those by Werner et al., report substantial variability in tibial tunnel positioning when using the LMAH as an intraoperative reference, with placements ranging from 26.4% to 49.2% along the anteroposterior axis of the tibial plateau [24]. Specifically, Werner et al. reported a mean sagittal placement of 37.0 ± 5.2% based on intraoperative fluoroscopic measurements, while Büyükdoğan et al. reported a mean sagittal placement of 39.3 ± 3.8% in their series of 60 patients [18,24].

In our study, the posterior border of the LMAH was similarly used as a reference during tibial tunnel placement; however, measurements were performed post-operatively using MRI. We found a mean AP tunnel position of 46.87%, which is more posterior compared to the values reported by Werner et al. and Büyükdoğan et al. [18,24]. The observed differences may be influenced not only by the imaging modality and timing of evaluation (post-operative MRI versus intraoperative fluoroscopy) but also by variability in surgical technique among different surgeons, such as differences in interpretation of landmarks, guide positioning, and drilling angles.

Using the LMAH as the primary tibial landmark may limit reliability when meniscal injury is present. Meniscal tears frequently accompany ACL rupture, particularly involving the lateral meniscus. In a large series of 1022 ACL-injured knees, Keyhani et al. found tears involving the posterior portion of the LMAH region (Cooper zone D3) in 1.8% of cases, and tears extending across the entire LMAH region (D1–D3) in 6.6% of cases [25]. In the present study, patients with concomitant meniscal tears were excluded to minimize this potential source of variability. When LMAH integrity, particularly its posterior margin, is compromised, combining it with other anatomical landmarks may improve accuracy and reproducibility.

The ACL most commonly tears at its mid-substance or avulses from the lateral femoral condyle; however, the tibial stump is typically left intact at surgery. This residual tissue often serves as a practical intraoperative reference for tibial tunnel placement, guiding surgeons in anatomic positioning. In the context of single-bundle ACL reconstruction, as performed in our study, accurate replication of the native tibial footprint remains critical for restoring functional knee kinematics [6].

However, despite its practical utility, the characteristics of the remnant may vary due to biological differences. Recent histologic data based on the epiligament (EL) theory suggest that the inconsistent appearance and robustness of the ACL remnant may be biologically driven. Comparative studies between the ACL and MCL ELs have demonstrated significantly lower vascularity and cellularity in the ACL, as well as regional heterogeneity within the ligament itself, which may influence remnant quality and healing potential [8,9,10,11]. These biological differences may explain intraoperative variability in remnant morphology and highlight the need for consistent, anatomy-based reference frameworks to ensure reproducible tunnel placement.

To enhance the consistency and anatomical accuracy of tibial tunnel placement, Siebold et al. [26] introduced the “tibial square model,” a landmark-based orientation method that defines the borders of the native ACL tibial footprint using identifiable arthroscopic references: the anterior and posterior margins of the ACL stump, the medial and lateral tibial condylar rims, and the posterior horn of the lateral meniscus. Although originally proposed for double-bundle techniques, this model also provides valuable guidance for single-bundle procedures by outlining a reproducible and individualized safe zone for tunnel positioning within the native footprint. By accommodating anteroposterior variability (14–15 mm in average insertion length), the model facilitates footprint-centric tunnel planning that can be tailored to patient-specific anatomy.

However, relying solely on the visible remnant may not ensure precise anatomical positioning. Pedneault et al. [6] reported that when the tibial stump was used as the exclusive reference, tunnel placement deviated an average of 6.24 mm from the native footprint, with only 45% achieving greater than 50% overlap. These findings highlight the limitations of remnant-based guidance and support the incorporation of objective anatomical frameworks such as the tibial square model even in single-bundle reconstructions, where personalized yet anatomically accurate tunnel placement remains essential.

Previous anatomical and radiological studies have reported comparable mediolateral localization of the ACL tibial footprint. Lee et al. described the anteromedial and posterolateral bundle centers at 47.1% and 53.5% along the mediolateral axis, respectively [27]. Similarly, Pietrini et al. found the centers at 44.2% and 50.1%, respectively [28]. Abreu-e-Silva et al. identified the tibial footprint center at 50.2% mediolaterally in their 3D CT-based single-bundle ACL reconstruction study [29]. Our mediolateral positioning results (47.99% for the ACL remnant group and 48.77% for the LMAH group) are therefore consistent with previously reported anatomical data, with no statistically significant difference observed between groups (*p* = 0.215).

Taken together, these results indicate that while the surgical reference may influence tunnel positioning in the sagittal plane, with a small mean difference of 2.3% (≈1.5 mm), it does not appear to impact clinical or functional outcomes. Both the ACL remnant and the LMAH can therefore be considered safe and reliable intraoperative landmarks for guiding tibial tunnel placement during ACL reconstruction.

To our knowledge, this is the first study to radiologically and clinically compare the ACL tibial remnant and LMAH as intraoperative landmarks for tibial tunnel placement in single-bundle ACL reconstruction. By integrating post-operative MRI-based anatomical assessment with clinical outcome measures, this dual-modality evaluation offers a comprehensive understanding of the practical reliability and anatomical precision of these commonly used reference points.

Several limitations should be acknowledged. First, the retrospective design introduces inherent risks of selection and information bias. Second, surgeries were performed by different surgeons. Although standardized techniques were nominally followed and all surgeons were experienced in ACL reconstruction, inter-surgeon variability—including differences in landmark interpretation, surgical experience, technical preferences (e.g., guide positioning, tunnel angle selection, and drilling approach), and surgical habits—may have influenced tunnel positioning. Additionally, while each surgeon predominantly adhered to a specific reference landmark, familiarity with other anatomical cues may have affected intraoperative decision-making. However, because landmark preference was entirely surgeon-dependent, statistical adjustment for surgeon effect was not feasible due to complete collinearity between surgeon and landmark choice. These factors could independently introduce bias related to surgical technique variability. Future analyses incorporating subgroup comparisons or regression adjustments for surgeon-related effects may help clarify their independent contribution. Third, although post-operative MRI provides accurate anatomical localization, it does not capture intraoperative nuances such as remnant quality, visualization challenges, or arthroscopic orientation, which can impact tunnel creation. Fourth, postoperative notch impingement and tunnel length parameters were not assessed in this study; however, their inclusion in future research would be valuable for a more comprehensive understanding of their potential relationship with tunnel positioning and graft behavior. Fifth, while the sample size was adequate for statistical analysis, the single-center nature and relatively limited cohort reduce the generalizability of the findings. Finally, the absence of long-term follow-up limits conclusions regarding the implications of tunnel positioning differences on graft integrity, joint stability, or degenerative progression.

Despite these limitations, this study offers important insights into the anatomical and clinical validity of tibial remnant- and LMAH-based tunnel placement strategies and underscores the importance of individualized, anatomy-oriented surgical planning.

## 5. Conclusions

In this study, both the ACL tibial remnant and the LMAH were confirmed as reliable intraoperative landmarks for tibial tunnel placement in single-bundle ACL reconstruction. The remnant group demonstrated a slightly more anterior sagittal tunnel position; however, this radiological difference did not affect short-term clinical or functional outcomes. These findings reflect short-term results and should not be generalized to long-term biomechanical or degenerative outcomes. Further prospective studies with extended follow-up are warranted to clarify the long-term implications of tunnel positioning.

## Figures and Tables

**Table 1:** Patient demographics.

	Group 1 (*n* = 35)	Group 2 (*n* = 39)	*p*
Age (years)	29.8 ± 7.9	27.7 ± 7.5	0.174 ^m^
Follow-up (months)	34.8 ± 12.2	33.74 ± 11.5	0.588 ^m^
Gender (M/F)	33/2	37/2	1.000 ^f^
BMI (kg/m^2^)	25.5 ± 2.3	25.0 ± 3.2	0.462 ^t^
Operated side (Right/Left)	15/20	23/16	0.166 ^X2^

BMI: body mass index. ^t^ independent samples *t*-test, ^X2^ chi-square test, ^m^ Mann–Whitney U test, ^f^ Fisher’s exact test.

**Table 2:** Preoperative and postoperative functional and activity scores.

	Group 1 (*n* = 35)	Group 2 (*n* = 39)	*p*
Tegner score			
Preinjury	7.29 ± 0.96	7.18 ± 0.94	0.409 ^m^
Preoperative	3.97 ± 1.04	4.08 ± 1.04	0.624 ^m^
Postoperative	6.49 ± 1.36	6.23 ± 1.69	0.568 ^m^
*p* ^†^ (overall)	8.50 × 10^−15 F^	3.28 × 10^−15 F^	
*p* ^†^ (Preinjury-Preoperative)	1.92 × 10^−7 w^	3.86 × 10^−8 w^	
*p* ^†^ (Preoperative-Postoperative)	3.04 × 10^−7 w^	2.15 × 10^−7 w^	
*p* ^†^ (Postoperative-Preinjury)	0.004 ^w^	0.0009 ^w^	
Lysholm score			
Preoperative	68.46 ± 7.92	68.64 ± 10.05	0.578 ^m^
Postoperative	86.37 ± 12.55	83.31 ± 13.67	0.191 ^m^
*p* ^†^	3.47 × 10^−7 w^	5.50 × 10^−8 w^	
IKDC score			
Preoperative	46.71 ± 9.60	45.49 ± 9.04	0.573 ^t^
Postoperative	83.26 ± 16.33	82.64 ± 16.36	0.637 ^m^
*p* ^†^	2.45 × 10^−7 w^	5.19 × 10^−8 w^	

^t^ independent samples *t*-test, ^m^ Mann–Whitney U test, ^w^ Wilcoxon signed-rank test, ^F^ Friedman test. ^†^ intragroup comparison.

**Table 3:** Comparison of knee stability parameters.

		Group 1 (*n* = 35)	Group 2 (*n* = 39)	*p*
Lachman test				
	Preoperative			
	Grade 1	6 (17.1%)	9 (23.1%)	0.587 ^m^
	Grade 2	21 (60.0%)	22 (56.4%)	
	Grade 3	8 (22.9%)	8 (20.5%)	
	Postoperative			
	Grade 0	27 (77.1%)	31 (79.5%)	0.856 ^m^
	Grade 1	8 (22.9%)	7 (17.9%)	
	Grade 2	0 (0.0%)	1 (2.6%)	
	*p* ^†^	4.39 × 10^−8 w^	1.66 × 10^−8 w^	
Pivot-shift test				
	Preoperative			
	Grade 1	7 (20.0%)	10 (25.6%)	0.496 ^m^
	Grade 2	18 (51.4%)	20 (51.3%)	
	Grade 3	10 (28.6%)	9 (23.1%)	
	Postoperative			
	Grade 0	29 (82.9%)	33 (84.6%)	0.799 ^m^
	Grade 1	5 (14.3%)	6 (15.4%)	
	Grade 2	1 (2.9%)	0 (0.0%)	
	*p* ^†^	8.65 × 10^−8 w^	2.69 × 10^−8 w^	
KT-2000 (mm) ^‡^	Mean ± SD	2.03 ± 1.48	2.18 ± 1.94	0.978 ^m^

^m^ Mann–Whitney U test, ^w^ Wilcoxon signed-rank test. ^†^ intragroup comparison, ^‡^ side-to-side difference.

**Table 4:** Comparison of return-to-sports parameters.

	Group 1 (*n* = 35)	Group 2 (*n* = 39)	*p*
Return to sports (Yes/No)	30/5	32/7	0.759 ^f^
Pivoting sports	23/5	25/7	
Non-pivoting sports	7/0	7/0	
Level of return to sports			
Lower level	4	6	0.733 ^f^
Same level	26	26	
Higher level	0	0	
Time to return to sports (months)	8.83 ± 2.17	8.88 ± 1.90	0.791 ^m^

^f^ Fisher’s exact test, ^m^ Mann–Whitney U test.

**Table 5:** Comparison of tunnel position.

	Group 1 (*n* = 35)	Group 2 (*n* = 39)	*p*
Sagittal Tunnel Position (%)	44.57 ± 4.85	46.87 ± 4.41	**0.036** ^t^
Anterior Cortex to Graft Center (mm)	24.69 ± 3.23	26.20 ± 2.93	**0.038** ^t^
Anterior Cortex to Posterior Cortex (mm)	55.39 ± 3.02	55.90 ± 3.81	0.466 ^t^
Coronal Tunnel Position (%)	47.99 ± 2.84	48.77 ± 2.68	0.215 ^m^
Medial Cortex to Graft Center (mm)	36.70 ± 2.57	37.50 ± 3.21	0.249 ^t^
Medial Cortex to Lateral Cortex (mm)	76.47 ± 3.54	76.89 ± 4.49	0.719 ^t^

^t^ independent samples *t*-test, ^m^ Mann–Whitney U test. Significant *p* values are written in bold.

## Data Availability

The data used and/or analyzed during the current study are available from the corresponding author upon reasonable request.

## Abbreviations

The following abbreviations are used in this manuscript:

ACL	Anterior cruciate ligament
BMI	Body mass index
ICC	Intraclass correlation coefficient
IKDC	International Knee Documentation Committee
LMAH	Lateral meniscus anterior horn
MTS	Medial tibial spine
MRI	Magnetic resonance imaging
SD	Standard deviation
